# Association of classroom-level stressors with psychological distress in teachers

**DOI:** 10.1093/occmed/kqae140

**Published:** 2025-01-17

**Authors:** D Titheradge, A Albajara Sáenz, R Hayes, O C Ukoumunne, T Ford

**Affiliations:** University of Exeter Medical School, University of Exeter, Exeter, UK; Department of Physiology, Pharmacology and Neuroscience, University of Bristol, Bristol, UK; Population Health Sciences, University of Bristol, Bristol, UK; Department of Psychiatry, University of Cambridge, Cambridge, UK; NIHR Applied Research Collaboration South West Peninsula, Department of Public Health and Sports Sciences, Faculty of Health and Life Sciences, University of Exeter, Exeter, UK; NIHR Applied Research Collaboration South West Peninsula, Department of Health and Community Sciences, Faculty of Health and Life Sciences, University of Exeter, Exeter, UK; University of Exeter Medical School, University of Exeter, Exeter, UK; Department of Psychiatry, University of Cambridge, Cambridge, UK

## Abstract

**Background:**

Poor mental health is highly prevalent among schoolteachers. Different occupational, contextual and personal factors have been identified as sources of their psychological distress.

**Aims:**

To explore the association of classroom-level variables with teachers’ mental health over the course of an academic year.

**Methods:**

This study included 80 primary schoolteachers and 2075 pupils from the STARS trial conducted in England, which explored the impact of the Incredible Years Teacher Classroom Management programme. Linear regression models examined the relationships between classroom-level predictor variables and teachers’ psychological distress, as measured by the Everyday Feeling Questionnaire, at 1 and 9 months into the school year. Predictor variables included classroom size and demographic composition, amount of teaching assistant support, and pupils’ mental health, as measured by the Strengths and Difficulties Questionnaire and the Pupil Behaviour Questionnaire. Analyses were adjusted for teacher length of service and trial arm status.

**Results:**

One month into the school year, fully adjusted analyses showed that having a classroom with a higher proportion of male pupils was associated with worse teacher mental health. None of the classroom-level stressors were associated with teacher mental health at 9 months.

**Conclusions:**

Classroom gender balance was associated with teacher’s mental health at the beginning but not at the end of the academic year. It is important to consider classroom-level variables when developing interventions and policies for teacher mental health.

## Teaser

This study examined the association of classroom-level factors (classroom size and demographic composition, teaching assistant support, pupils’ mental health) with teachers’ mental health over an academic year, with 80 primary school teachers and 2075 pupils in England participating. A higher proportion of male pupils in the classroom was associated with worse teacher mental health early in the school year, highlighting the potential importance of classroom composition for teacher mental health.

## Introduction

Teachers are at a high risk of experiencing poor mental health, with reports of higher rates of work-related stress, depression and anxiety compared with other occupations [1–3]. Poor mental health in teachers is associated with negative personal and professional outcomes, including high attrition, absenteeism, presenteeism, lower occupational commitment, job satisfaction and self-efficacy [1,4–7]. Additionally, poor teacher mental health adversely impacts students’ academic achievement, learning and mental health, and teacher–student relationships [8–10].

Different individual and contextual factors contribute to poor teacher mental health [6,11]. Occupational factors include an excessive workload, frequently changing government requirements, low salaries, limited professional development, and addressing stakeholder requests and special educational needs (SEN) [6,12]. Teachers in publicly funded, urban schools with a high concentration of disadvantaged students face an elevated risk of stress [6]. Classroom-level factors, such as class size and demographic composition, also impact teacher mental health. Lower socio-economic status in children is associated with mental health and behavioural difficulties, educational failure and SEN [13–15]. Challenging student behaviour is one of the main classroom-related sources of stress for teachers [6,7,16]. There are marked gender differences in children regarding academic performance, mental health and behavioural difficulties, with conditions like attention-deficit/hyperactivity disorder (ADHD) and conduct disorder being more prevalent in boys [17–19]. Although teachers with a higher number of students experience higher workload and classroom management demands, studies on class size and teacher stress report mixed results [20,21], perhaps because some of these challenges may be offset by the presence of classroom support [11].

Examining classroom-level factors affecting teachers’ mental health can help identify pragmatic strategies and interventions to address the increased burden of poor mental health in teachers and the subsequent consequences for students. However, most research has focused on student behaviour or studied factors separately, often cross-sectionally. This study aimed to explore the association of various classroom characteristics with teachers’ psychological distress over the course of an academic year. Associations with teachers’ mental health at both the beginning and end of the academic year were assessed to determine whether this changed. Based on previous literature, we hypothesized that a larger class size, a higher proportion of male or socio-economically disadvantaged students, students with SEN, poor pupil behaviour and mental health, and limited classroom support, would be associated with poorer teacher mental health. Data from the STARS (Supporting Teachers And childRen in Schools) trial, a randomized controlled trial exploring the effects of the Incredible Years Teacher Classroom Management (TCM) training on pupil mental health, was used in this study [22].

## Methods

The STARS trial (registration: SRCTN84130388) received ethical approval from the Peninsula College of Medicine and Dentistry Research Ethics Committee, now under the University of Exeter Medical School Committee (reference: 12/03/14).

Participants were 80 primary schoolteachers from 80 different state-funded schools in the South West of England, and 2075 pupils from their classes, participating in the STARS trial. In the trial, the unit of allocation was the school, with one teacher per school participating in the study, to prevent contamination and reflect real practice intervention roll-out [22]. Teachers had classroom responsibility for a single-year group at least 4 days per week, with a minimum 3-year contract to cover trial data collection. Classes required 15 or more pupils from Reception to Year 4, aged 4–9 years at recruitment. Schools were excluded if they primarily taught pupils with SEN, lacked a substantive head teacher or were judged as failing in their last Ofsted (Office for Standards in Education, Children’s Services and Skills in England) inspection. Headteachers consented to their school’s participation and, without any given criteria, nominated a teacher who consented to their own participation. Parents and children with insufficient English to understand recruitment information and complete outcome measures were excluded. Parents were sent information about the study and were provided with 2 weeks to opt their children out of it. Further information regarding the sample size can be found in the STARS trial report [22].

**Figure 1.**
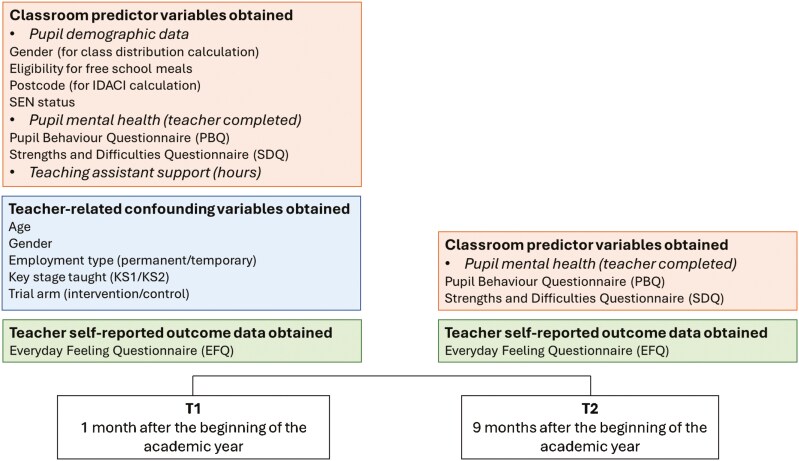
Overview of study design and data collection. IDACI = Income Deprivation Affecting Children Index; SEN = Special Educational Needs, defined by pupils at a School Action level or higher.

The main outcome variable, the Everyday Feeling Questionnaire (EFQ), was completed by teachers approximately 1 month into the school year and after approximately 9 months of teaching the class, in June/July (Figure 1). The EFQ is a 10-item self-reported measure of psychological well-being and distress over the previous 4 weeks [23,24]. Half of the items focus on psychological distress (scored 0–4), while the other half focus on well-being (scored 4–0). The possible total score ranges from 0 to 40, with a higher score indicating increased distress and reduced well-being, and a score above 19 indicating at least moderate levels of clinical depression [2,25].

Pupils’ socio-demographic predictor variables included gender and social deprivation, provided by teachers and parents at baseline. Classes with a balanced gender distribution (45–55% males) were compared against those with greater than 55% males or females. Social deprivation was measured by the Income Deprivation Affecting Children Index (IDACI), based on the school’s postcode [26]. The IDACI is the proportion of children aged 0–15 living in income-deprived households, ranging from 0 to 1 (i.e. 0–100%). The IDACI was dichotomized to compare schools in the highest deprivation quintile-based group (ranging from 0.30 to 0.48) with other schools (ranging from 0.03 to 0.28). The percentage of pupils receiving free school meals (FSM) in each class, an additional measure of socio-economic deprivation, was dichotomized into classes with less than 19% (low deprivation) and classes with 19% or more (high deprivation) of pupils receiving FSM; 19% was the UK national average in 2012 when baseline data were collected [27]. The percentage of students requiring SEN support was used, covering those with School Action, School Action Plus, Statements of SEN, or Education, Health and Care Plans. Classroom teaching assistant support ranged from no additional support (0 hours/week), part-time support (1–24 hours/week), to full-time support (25 hours/week).

Pupil mental health predictor variables included the Pupil Behaviour Questionnaire (PBQ) and the Strengths and Difficulties Questionnaire (SDQ), completed by teachers at baseline and after 9 months. Class mean scores indicated the level of psychopathology reported by each teacher. The PBQ is a six-item questionnaire measuring classroom-based disruptive behaviours, with the response options: never (0), occasionally (1) and frequently (2). The total score ranges from a possible 0 to 12, with a higher score indicating more disruptive behaviour [28]. The SDQ is a 25-item behavioural screening questionnaire with five subscales: Emotional Symptoms, Conduct Problems, Hyperactivity/Inattention, Peer Relationship Problems, and Prosocial Behaviour [29]. Each item is rated on a 3-point scale (0 = Not True; 1 = Somewhat True; 2 = Certainly True). Responses to the 20 items on the first four subscales are summed to give a Total Difficulties score (ranging from a possible 0 to 40), with higher scores indicating poorer mental health [30]. The Impact Supplement contains three items exploring whether the teacher believes that the child has a problem and covers the impact of a pupil’s difficulties on classroom learning and peer relationships, and whether the pupil is a burden on the class [31]. Response options include: ‘Not at all’ and ‘Only a little’ (0), ‘A medium amount’ (1), and ‘A great deal’ (2). Possible scores for teacher-rated SDQ Impact range from a possible 0 to 6.

Potential confounding variables included teachers’ length of service, gender, appointment type (permanent, temporary/probationary), and key stage (KS) taught (KS1: 4- to 6-year olds; KS2: 7- to 11-year olds). Teacher age was not considered for the analysis due to high collinearity with teacher length of service. Of these, only teacher length of service was a statistically significant predictor of EFQ and was included as a confounder in adjusted analyses. Although the STARS trial showed no effect of the TCM programme on teacher mental health, there were small but statistically significant effects on student mental health, so trial arm status was also included as a confounder in adjusted analyses [25,32].

Statistical analysis was conducted using R version 3.5.3 [33]. Descriptive statistics for confounder, predictor and outcome variables at the school, teacher and class levels were reported. Pearson’s correlation coefficient was used to quantify the association amongst quantitative variables. Paired *t*-tests were used to examine changes in EFQ, SDQ, SDQ Impact, and PBQ between 1 and 9 months.

Linear regression was used to examine the relationship between predictor variables (class size, % boys, % pupils with SEN, classroom support hours, IDACI, SDQ, SDQ Impact and PBQ) and EFQ at each of 1 and 9 months. Pupil mental health predictors (SDQ, SDQ Impact, PBQ) measured at 1 and 9 months were used in the analysis for EFQ at 1 and 9 months, respectively, to quantify cross-sectional associations for those variables. First, predictors were included separately in unadjusted (crude) simple linear regression models for each time point (Model 1). All predictors significantly associated with EFQ were included simultaneously in subsequent models that were adjusted for teacher length of service and trial arm status. Where predictors were highly correlated, separate adjusted regression models were fitted for each of the correlated variables (Model 2). Finally, all statistically significant predictors were included in a single fully adjusted model (Model 3). Regression diagnostics were performed and assumptions for linear regression were met throughout.

## Results

Response rates were high for teacher-reported pupil data at baseline (100%, *n* = 2074 children) and 9 months (96%, *n* = 2000 children), and for teacher self-report at 1 month (100%; *n* = 80) and 9 months (93%, *n* = 74). Six teachers were lost to follow-up due to leaving the school, sick leave or maternity leave. Teacher and pupil demographics and classroom and school-level data are summarized in Table 1. Teachers were predominantly female (80%) and evenly distributed across KS1 and KS2; 54% of schools were urban and 46% semi-rural/rural. The mean (SD; range) teaching experience was 6.7 years (6.2; 0–23). There were 1101 boys (53%) and 974 girls (47%), with 19% of pupils having SEN.

**Table 1.** School, teacher, classroom and pupil demographics

Demographics		*n* (%)
School demographics (*N* = 80)		
Percentage of free school meals above 19%		31 (39)
IDACI decile by school postcode	Most deprived quintile-based group	16 (20)
	Other groups	64 (80)
School setting	Rural	37 (46)
	Urban	43 (54)
Trial arm	Control	40 (50)
	Intervention	40 (50)
Teacher demographics (*N* = 80)		
Teacher gender	Male	15 (19)
	Female	65 (81)
Key stage taught	Key stage 1	41 (51)
	Key stage 2	39 (49)
Employment type	Permanent	66 (83)
	Temporary	14 (18)
Classroom-level demographics (*N* = 80)		
Gender balance	Balanced (45–55% male)	27 (34)
	>55% Male	38 (48)
	>55% Female	15 (19)
Free school meals	Less than 19%	49 (61)
	19% or more	31 (39)
Pupil demographics (*N* = 2075)		
Pupil gender	Male	1101 (53)
	Female	974 (47)
Pupils with SEN		392 (19)

IDACI = Income Deprivation Affecting Children Index; SEN = Special Educational Needs, defined by pupils at a School Action level or higher.

Teacher EFQ data at 1 and 9 months are reported in Table 2. Teachers had a wide range of class sizes and composition, in terms of socio-economic status, number of children with SEN and classroom support. Correlations among quantitative predictors, confounders and outcomes are reported in Table 1 (available as Supplementary data at *Occupational Medicine* Online).

**Table 2.** Descriptive statistics for outcome and predictor variables

Class/teacher characteristic			Mean (SD)	Range
Outcome variables	Teacher-rated	EFQ at 1 month	15.6 (6.9)	2–33
		EFQ at 9 months	14.6 (6.9)	0–30
Predictor variables	Class demographics	Class size	25.9 (3.5)	17–33
		% Pupils with SEN	19.3 (11.1)	0–46
		Classroom support hours	20.0 (7.3)	0–25
	Teacher-rated (class level means)	Pupil Behaviour Questionnaire at 1 month	1.9 (0.8)	0.4–3.8
		Pupil Behaviour Questionnaire at 9 months	1.8 (0.9)	0.4–4.4
		SDQ Total Difficulties score at 1 month	6.7 (2.3)	2.8–12.9
		SDQ Total Difficulties score at 9 months	6.0 (2.7)	1.1–16
		SDQ Impact Clinical score at 1 month	0.4 (0.3)	0–1.2
		SDQ Impact Clinical score at 9 months	0.4 (0.3)	0–1.5
Confounding variables	Teacher demographics	Teacher age	32.9 (8.9)	22–56
		Teacher length of service	6.7 (6.2)	0–23

EFQ = Everyday Feelings Questionnaire; SDQ = Strengths and Difficulties Questionnaire; SEN = Special Educational Needs, defined by pupils at a School Action level or higher.

No statistically significant changes in EFQ, SDQ Impact and PBQ were observed between 1 and 9 months, but a significant decrease in SDQ was found (*P* < 0.01).

Regression analyses that examined the predictors in separate unadjusted models (Model 1) identified statistically significant associations for each of classroom gender balance, mean class SDQ and PBQ with teacher EFQ at 1 month (Table 3). These were used as predictors simultaneously in the subsequent multivariable regression models that were adjusted for teacher length of service and trial arm. Due to the correlation between SDQ and PBQ (Pearson’s *r* = 0.62), models including gender balance and mean class SDQ and gender balance and mean class PBQ were run separately (Models 2a and 2b). There were statistically significant associations of mean class SDQ and mean class PBQ with EFQ at 1 month in the respective models, and an association of gender balance in both models. In the final full multivariable model with both mean class SDQ and mean class PBQ, only gender balance retained statistical significance (Model 3).

**Table 3.** Linear regression analyses of EFQ at 1 month (outcome) on predictors

Predictor		Unadjusted regression of EFQ at 1 month (Model 1)		Adjusted regression of EFQ at 1 month (Model 2a)		Adjusted regression of EFQ at 1 month (Model 2b)		Fully adjusted regression of EFQ at 1 month (Model 3)	
		Coefficient	p	Coefficient	p	Coefficient	*P*	Coefficient	*P*
		(95% CI)		(95% CI)		(95% CI)		(95% CI)	
Class Size		−0.0 (−0.5 to 0.4)	1	-	-	-	-	-	-
Gender balance	Balanced (45% to 55% Boys)	**Reference**	**0.05**	**Reference**	**0.03**	**Reference**	**0.03**	**Reference**	**0.03**
	>55% Boys	**4.1 (0.7 to 7.4)**		**4.38 (1.3 to 7.5)**		**4.06 (0.9 to 7.3)**		**4.22 (1.1 to 7.4)**	
	>55% Girls	**1.0 (−3.4 to 5.3)**		**1.86 (−2.1 to 5.8)**		**2.07 (−2 to 6.1)**		**1.97 (−2.1 to 6)**	
Percentage SEN (per 10% increase)		0.0 (−1.4 to 1.4)	0.99	-	-	-	-	-	-
High deprivation (IDACI lowest Quintile)		1.2 (−2.7 to 5.1)	0.54	-	-	-	-	-	-
High deprivation (Free school meals)		0.3 (−2.9 to 3.4)	0.87	-	-	-	-	-	-
Mean class SDQ at 1 month		**0.9 (0.3 to 1.6)**	**0.005**	**0.83 (0.2 to 1.4)**	**0.01**	-	-	0.69 (−0.1 to 1.4)	0.07
Mean class SDQ Impact at 1 month		3.8 (−1.6 to 9.3)	0.16	-	-	-	-	-	-
Mean class PBQ at 1 month		**2.5 (0.6 to 4.5)**	**0.01**	-	-	**1.99 (0.1 to 3.9)**	**0.04**	0.73 (−1.6 to 3)	0.53
Classroom support hours		0.0 (−0.2 to 0.2)	0.9	-	-	-	-	-	-
Teacher length of service		N/A	N/A	**0.23 (0 to 0.5)**	**0.05**	0.23 (0 to 0.5)	0.05	0.23 (0 to 0.5)	0.05
School allocation (intervention)		N/A	N/A	**3.07 (0.3 to 5.9)**	**0.03**	**3.08 (0.2 to 5.9)**	**0.03**	**3.04 (0.2 to 5.8)**	**0.03**

EFQ = Everyday Feelings Questionnaire; PBQ = Pupil Behaviour Questionnaire; SDQ = Strengths and Difficulties Questionnaire; SEN = Special Educational Needs, defined by pupils at a School Action level or higher; IDACI = Income Deprivation Affecting Children Index.

Unadjusted regression analyses that examined the predictors in separate models (Model 1) identified statistically significant associations for each of mean class SDQ and mean class SDQ Impact scores with EFQ at 9 months (Table 4). Following adjustment for teacher length of service and trial arm status, both mean SDQ and mean SDQ Impact scores retained statistical significance as predictors of EFQ at 9 months (Models 2a and 2b, respectively). In the final full multivariable analysis, none of the main potential predictors of interest were statistically significant (Model 3).

**Table 4.** Linear regression analyses of EFQ at 9 months (outcome) on predictors

Predictor		Unadjusted regression of EFQ at 9 months (Model 1)		Adjusted regression of EFQ at 9 months (Model 2a)		Adjusted regression of EFQ at 9 months (Model 2b)		Fully adjusted regression of EFQ at 9 months (Model 3)	
		Coefficient	p	Coefficient	p	Coefficient	p	Coefficient	P
		(95% CI)		(95% CI)		(95% CI)		(95% CI)	
Class Size		−0.2 (−0.7 to 0.2)	0.36	–	–	–	–	–	–
Gender balance	Balanced (45% to 55% Boys)	Reference	0.10	–	–	–	–	–	–
	>55% Boys	3.3 (−0.3 to 6.8)		–	–	–	–	–	–
	>55% Girls	−0.3 (−4.8 to 4.2)		–	–	–	–	–	–
Percentage SEN (per 10% increase)		0.3 (−1.1 to 1.7)	0.69	–	–	–	–	–	–
High deprivation (IDACI lowest Quintile)		3.6 (−0.3 to 7.5)	0.07	–	–	–	–	–	–
High deprivation (Free school meals)		−0.6 (−3.9 to 2.6)	0.70	–	–	–	–	–	–
Mean class SDQ at 9 months		**0.64 (0 to 1.2)**	**0.04**	**0.75 (0.2 to 1.3)**	**0.01**	–	–	0.52 (−0.3 to 1.4)	0.23
Mean class SDQ Impact at 9 months		**5.22 (0.1 to 10.3)**	**0.05**	–	–	**5.84 (1 to 10.7)**	**0.02**	2.54 (−4.8 to 9.9)	0.49
Mean class PBQ at 9 months		1.49 (−0.4 to 3.4)	0.12	–	–	–	–	–	–
Classroom support hours		-0.1 (-0.3 to 0.2)	0.62	–	–	–	–	–	–
Teacher length of service		N/A	N/A	**0.36 (0.1 to 0.6)**	**0.01**	**0.34 (0.1 to 0.6)**	**0.01**	0.35 (0.1 to 0.6)	**0.01**
School allocation (intervention)		N/A	N/A	1.81 (−1.2 to 4.8)	0.24	1.73 (−1.3 to 4.8)	0.26	1.83 (−1.2 to 4.9)	0.23

EFQ = Everyday Feelings Questionnaire; IDACI = Income Deprivation Affecting Children Index; PBQ = Pupil Behaviour Questionnaire; SDQ = Strengths and Difficulties Questionnaire; SEN = Special Educational Needs, defined by pupils at a School Action level or higher.

## Discussion

This study showed an association between classroom gender balance and school teachers’ mental health 1 month into the academic year. Statistical power was limited by the sample size and these relationships should be investigated further, using a larger sample of teachers. The PBQ and SDQ measures, rated by teachers, may have been influenced by their mental health at the time, suggesting potential reporter bias, that warrants further investigation. Additionally, it is possible that headteachers selected teachers for participation who were either struggling with behaviour management or had a particular interest in it, potentially introducing bias. However, interviews with headteachers revealed a variety of reasons for their nominations [22,25], possibly mitigating this risk.

First, fully adjusted analyses showed that 1 month into the academic year, teaching a class with a higher proportion of male pupils was associated with worse teacher mental health. Boys are more likely to experience behavioural issues, with a higher prevalence of ADHD and conduct disorders [34,35]. This finding highlights the importance of classroom composition in teacher mental health. By 9 months into the academic year, the confounding variable longer length of service was associated with higher psychological distress, consistent with reports of lower occupational well-being in more experienced teachers [12], but contrasting with studies showing higher distress in less experienced teachers [36]. Different mental health constructs may be affected in experienced and novice teachers, with novice teachers experiencing higher levels of burnout, and more experienced teachers reporting higher exhaustion and stress, related to perceiving their profession as less valued by society, salary dissatisfaction and administrative burden [6,36]. The difference in results at 1 and 9 months may reflect the initial challenges of settling into a class with a higher proportion of male pupils and potentially greater levels of disruptive behaviour. Over time, teachers may have adjusted to classroom dynamics and developed behaviour management strategies, including the ones from the TCM programme in the intervention group, diluting the impact of classroom gender balance on teacher mental health by 9 months. Later in the academic year, length of service becomes more relevant, possibly due to the cumulative effects of long-term stressors associated with a longer teaching career.

Partially adjusted analyses showed an association between pupils’ mental health and behaviour and teacher mental health. Existing literature supports disruptive classroom behaviour as a major source of teacher burnout and work-related stress [6,16]. Both SDQ and PBQ scores were no longer statistically significant predictors in the fully adjusted model at one month due to their collinearity. At 9 months, SDQ and SDQ Impact were both significant predictors of teacher mental health in partially adjusted models but not in the fully adjusted model, again likely due to their overlapping nature. Our results highlight the need for further research exploring the impact of pupil behaviour and mental health on teacher mental health.

Class size, SEN proportion, socio-economic deprivation and classroom support did not have statistically significant associations with teacher mental health at either time point. Although recent research has shown increased levels of burnout in teachers with a higher number of students requiring support [11], different SEN types might impact teacher mental health differently, with disruptive behaviour being more detrimental to teachers’ mental health and attitudes than communication or physical difficulties [37,38]. Combining different SEN types might have masked differential associations between certain types of SEN and teacher psychological distress. Another possibility is that support for pupils with SEN is working efficiently, reducing the burden of SEN. Additionally, the lack of association between socio-economic deprivation and teacher mental health in our study aligns with Temam *et al*., who found no statistically significant differences in work-related well-being between teachers in schools with higher versus lower proportions of disadvantaged students [39]. When teachers perceive that available resources meet classroom demands, they are less likely to experience poor mental health [7]. In our study, available resources may have compensated for the demands associated with students with SEN, although teachers’ perceptions of the balance between demands and available resources were not assessed.

Teachers’ psychological distress, measured by the EFQ, was lower than in clinical populations, but higher than in the general population [2], indicating raised and sustained distress among this cohort of teachers and consistent with previous reports of poor teacher mental health [1]. EFQ scores also showed high variability in teachers’ distress over time, possibly influenced by factors not investigated in this study.

In conclusion, this small-scale study suggests classroom gender balance is associated with teachers’ mental health early in the school year, highlighting the importance of considering classroom-level variables in interventions and policies to support their mental health. The high prevalence and burden of distress among teachers calls for targeted mental health interventions, with particular attention to more experienced teachers and recognition of classroom composition as an important factor in teacher well-being.

**Key learning points**

**What is already known about this subject:**

- Teachers experience poorer mental health compared to other occupational groups with negative consequences for teachers themselves personally and professionally and for their pupils.
- Different occupational, contextual and personal factors have been associated with teachers’ psychological distress.
- However, research on classroom factors related to teachers’ mental health has generally focused on pupil behaviour or studied explanatory factors separately.

**What this study adds:**

- Teachers’ psychological distress, measured by the EFQ, was lower than in clinical populations, but higher than in the general population.
- One month into the school year, fully adjusted analyses showed that having a classroom with a higher proportion of male pupils was associated with poorer teacher mental health.
- Class size, SEN proportion, socio-economic deprivation and classroom support did not have statistically significant associations with teacher mental health.

**What impact this may have on practice or policy:**

- Classroom composition is a potentially important factor in teacher mental health and well-being.
- Enhanced support for more experienced teachers may be necessary to address their unique mental health challenges.

## Supplementary Material

kqae140_Supplementary_Table_S1
