# Supplementary material for: Association of classroom-level stressors with psychological distress in teachers
Source: Occup Med (Lond). 2025 Jan 17;75(6):324–31. doi: 10.1093/occmed/kqae140 (PMC12449249; doi:10.1093/occmed/kqae140)
Supplement: kqae140_Supplementary_Table_S1 [file kqae140_supplementary_table_s1.doc]

## **Additional Table 1: Pearson correlation coefficients between continuous** variables

|  | | Class Size | Percentage SEN | Mean Class SDQ | | Mean Class SDQ Impact Clinical | | Mean Class PBQ | | Classroom Support Hours | Teacher Age | Teacher Length of Service | EFQ | |
| --- | --- | --- | --- | --- | --- | --- | --- | --- | --- | --- | --- | --- | --- | --- |
|  |  |  |  | 1 month | 9 months | 1 month | 9 months | 1 month | 9 months |  |  |  | 1 month | 9 months |
| Class Size | | 1 | -0.28 | -0.13 | -0.19 | -0.02 | -0.04 | -0.06 | -0.24 | 0.06 | 0.1 | 0.1 | -0.02 | -0.11 |
| Percentage SEN | | -0.28 | 1 | 0.06 | 0.26 | 0 | 0.11 | 0.13 | 0.25 | -0.09 | -0.14 | -0.11 | -0.04 | 0.05 |
| Mean Class SDQ | 1 month | -0.13 | 0.06 | 1 | 0.66 | 0.61 | 0.54 | 0.62 | 0.44 | 0.17 | 0.18 | 0.13 | 0.36 | 0.25 |
|  | 9 months | -0.19 | 0.26 | 0.66 | 1 | 0.34 | 0.75 | 0.41 | 0.72 | 0.23 | -0.03 | -0.08 | 0.27 | 0.24 |
| Mean Class SDQ Impact | 1 month | -0.02 | 0 | 0.61 | 0.34 | 1 | 0.53 | 0.52 | 0.24 | 0.19 | 0.21 | 0.2 | 0.15 | 0.06 |
|  | 9 months | -0.04 | 0.11 | 0.54 | 0.75 | 0.53 | 1 | 0.48 | 0.66 | 0.11 | -0.02 | -0.05 | 0.17 | 0.24 |
| Mean Class PBQ | 1 month | -0.06 | 0.13 | 0.62 | 0.41 | 0.52 | 0.48 | 1 | 0.61 | 0.16 | 0.09 | 0.01 | 0.29 | 0.14 |
|  | 9 months | -0.24 | 0.25 | 0.44 | 0.72 | 0.24 | 0.66 | 0.61 | 1 | 0.14 | 0.01 | -0.05 | 0.21 | 0.18 |
| Classroom Support Hours | | 0.06 | -0.09 | 0.17 | 0.23 | 0.19 | 0.11 | 0.16 | 0.14 | 1 | 0.26 | 0.19 | -0.02 | -0.06 |
| Teacher Age | | 0.1 | -0.14 | 0.18 | -0.03 | 0.21 | -0.02 | 0.09 | 0.01 | 0.26 | 1 | 0.72 | 0.29 | 0.28 |
| Teacher Length of Service | | 0.1 | -0.11 | 0.13 | -0.08 | 0.2 | -0.05 | 0.01 | -0.05 | 0.19 | 0.72 | 1 | 0.28 | 0.3 |
| EFQ | 1 month | -0.02 | -0.04 | 0.36 | 0.27 | 0.15 | 0.17 | 0.29 | 0.21 | -0.02 | 0.29 | 0.28 | 1 | 0.69 |
| EFQ | 9 months | -0.11 | 0.05 | 0.25 | 0.24 | 0.06 | 0.24 | 0.14 | 0.18 | -0.06 | 0.28 | 0.3 | 0.69 | 1 |

EFQ = Everyday Feelings Questionnaire; PBQ = Pupil Behaviour Questionnaire; SDQ = Strengths and Difficulties Questionnaire; SEN = Special Educational Needs, defined by pupils at a School Action level or higher.
